# Transthoracic echocardiography and cardiac biomarkers in healthy captive male and female squirrel monkeys (*Saimiri spp.*)

**DOI:** 10.1186/s12917-020-02406-3

**Published:** 2020-06-29

**Authors:** Laurent Locquet, Blandine Houdellier, Bart J. G. Broeckx, Tim Bouts, Veronique Liekens, Jimmy H. Saunders, Pascale Smets

**Affiliations:** 1 Small Animal Department, Faculty of Veterinary Medicine, Ghent University, Merelbeke, Belgium; 2 Department of Medical Imaging and Small Animal Orthopedics, Faculty of Veterinary Medicine, Ghent University, Merelbeke, Belgium; 3 Department of Nutrition, Genetics and Ethology, Faculty of Veterinary Medicine, Ghent University, Merelbeke, Belgium; 4 Pairi Daiza, Brugelette, Belgium

**Keywords:** Squirrel monkey, Saimiri, Echocardiography, Cardiac troponin T, NT-proBNP

## Abstract

**Background:**

Echocardiography is the most frequently used non -invasive diagnostic tool to evaluate cardiac anatomy and function in domestic species but increasingly also in non -domestic species, especially since cardiac disease is being recognized as an important cause of death in captive primates. The purpose of this cross -sectional study was to investigate the feasibility of transthoracic echocardiography in healthy squirrel monkeys as well as to provide species specific normal values for standard echocardiographic measurements. A secondary aim was to determine plasma and serum levels of the cardiac biomarkers, N -terminal pro -brain natriuretic peptide (NT -proBNP) and cardiac troponin T (cTnT). Furthermore, a commercial, non -invasive, smartphone -based ECG (AliveCor Vet TM) monitoring device was used to evaluate the heart rate and rhythm and to diagnose possible arrhythmias.

**Results:**

In this study, transthoracic echocardiography of 14 squirrel monkeys was performed in right and left lateral recumbency. Similar standard right parasternal and left apical images were obtained as in dogs and cats and normal values for routine two -dimensional, time motion mode and Doppler mode measurements were generated. Thirteen animals were considered healthy and one squirrel monkey was identified with significant aortic dilation and regurgitation and consequently values obtained from this animal were not used when species specific normal values were calculated. NT -ProBNP and cTnT concentrations were available for 7 of the 13 healthy monkeys with NT -proBNP concentrations below detection limit in all animals and a mean cTnT concentration of 0.049 ng/mL. Electrocardiography was performed in all squirrel monkeys. The mean heart rate was 172 bpm. Frequent supraventricular premature beats were diagnosed in the squirrel monkey suffering from significant aortic dilation and regurgitation.

**Conclusion:**

This study presents echocardiographic normal values and additional cardiovascular data in anaesthetised Saimiri monkeys, fundamental from both the perspective of zoo animal health care as well as scientific research, since the squirrel monkey is often used as an animal model for human disease.

## Background

Squirrel monkeys (family Cebidae, subfamily Saimirinae) are among the most commonly studied animal models in biomedical research, both in veterinary and human medicine, the latter due to their phylogenetic similarities [1]. These arboreal neotropical nonhuman primates have been subdivided into divergent taxa on which numerous biological, biogeographical, morphological and behavioural studies have been published in the past [2–11]. Despite these earlier studies, vital information for both wildlife conservation, zoological management and future biomedical research is still lacking, indicating the importance of additional studies in order to better understand the biology and morphology of the squirrel monkey.

Several studies in both human and nonhuman primates have identified cardiovascular disease as a major cause of death with mortality rates ranging from 41% in gorillas to approximately 81% in chimpanzees (Meehan and Lowenstine, 1994 [12–15];). Earlier studies and case reports have described one or multiple cases of dilated, hypertrophic (-like) and fibrosing cardiomyopathy in several monkey species, including the Rhesus macaque (*Macaca mulatta*), owl monkey (*Aotus* sp.) and squirrel monkey [12, 13, 15–24].

Given the significant mortality secondary to cardiovascular disease, previous research has concentrated on thoracic radiographic, electrocardiographic and echocardiographic characteristics of squirrel monkeys [25, 26]. However, only few echocardiographic parameters have been described earlier and an echocardiographic protocol is currently lacking in the squirrel monkey.

Echocardiography is the most commonly used diagnostic tool for non -invasive assessment of cardiac structures and function. Elevated blood concentrations of cardiac biomarkers such as N -terminal pro -brain natriuretic peptide (NT -proBNP), cardiac troponin I (cTnI) and T (cTnT) are associated with several types of cardiac disease in human and veterinary medicine [27–30]. Cardiac troponin T is an intracellular protein, bound to the actin backbone within cardiomyocytes. Elevated cTnT values, indicating recent damage of cardiomyocytes, can be a useful diagnostic piece of a puzzle in cardiac disease in both domestic and non domestic mammals [27]. Given the fact that cardiac troponin is encoded in higher vertebrates by orthologous genes, this biomarker appears to be highly conserved across species [31, 32]. Increased NT -proBNP concentrations are associated with myocardial stretch, as described in many human and veterinary studies [33].

Although an echocardiographic protocol in great apes has been described and is currently being applied in a large international study in chimpanzees, gorillas, bonobos and orangutans, no such protocol has been defined in the squirrel monkey [34–38]. The general protocol applied in great apes is comparable to echocardiography performed in humans, as the anatomy and morphology of the thorax is similar. Given a similar thoracic morphology compared to cats and dogs, it was decided to follow the protocol for feline and canine echocardiographic examinations, which has been thoroughly described [39, 40].

The primary aims of this study were to test the feasibility of transthoracic echocardiography (TTE), following a protocol similar to canine and feline echocardiography, in healthy squirrel monkeys as well as to provide species specific normal values for standard echocardiographic measurements. A secondary aim was to determine blood concentrations of two cardiac biomarkers, NT -proBNP and cTnT, and to evaluate heart rate and rhythm using a non -invasive, smartphone based ECG (AliveCor Vet TM) monitoring device.

## Results

One *Saimiri boliviensis peruviensis* presented with a significant abnormality (severe dilation of the ascending aorta with aortic regurgitation) on echocardiography and arrhythmia (frequent supraventricular beats) on electrocardiography and was excluded from the study. The age of the 13 remaining animals ranged from 2.25 to 21.3 years (mean age = 9.12 years ± SD of 6.95 years). The minimum and maximum weights of the animals were 467 g and 756 g respectively (mean weight = 615 g ± SD of 92 g). There were 11 females and 2 males.

Good quality echocardiographic examinations were obtained in all 13 healthy animals and 25 variables were measured (Table 1). Eleven squirrel monkeys (*Saimiri* spp.) showed signs of trace valvular insufficiency without clinical relevance, namely of the mitral valve (n = 2), tricuspid valve (n = 6), aortic valve (n = 2) and pulmonic valve (n = 7). Furthermore, one mild mitral valve insufficiency was diagnosed without signs of cardiac remodelling.

**Table 1:** Mean ± SD, minimum and maximum values (range) of measurements of echocardiographic parameters obtained in 13 captive squirrel monkeys (Saimiri spp.) from transthoracic echocardiographic examinations using 2D -mode, M -mode and Doppler mode

	Echocardiographic parameter	Mean ± SD	Range
2D	IVSd (mm)	2.90 ± 0.45	2.24 -3.60
	LVIDd (mm)	9.38 ± 0.74	7.74 -10.38
	LVPWd (mm)	2.79 ± 0.32	2.38 -3.383.42
	IVSs (mm)	4.14 ± 0.67	3.05 -5.29
	LVIDs (mm)	5.30 ± 0.77	43.92 -6.36
	LVPWs (mm)	4.49 ± 0.63	3.25 -5.62
	FS (%)	44 ± 5	36 -52
	Ao (mm)	4.56 ± 0.52	4.06 -5.95
	LAD (mm)	6.47 ± 0.60	5.60 -7.62
	LA/Ao	1.42 ± 0.06	1.28 -1.50
	LALAX (mm)	7.85 ± 0.84	6.51 -9.62
	RALAX (mm)	6.56 ± 0.98	5.52 -9.31
M -mode	IVSd (mm)	2.90 ± 0.52	2.19 4.04
	LVDd (mm)	9.15 ± 0.90	7.59 -10.41
	LVPWd (mm)	2.97 ± 0.47	2.12 -3.78
	IVSs (mm)	4.15 ± 0.67	3.06 -5.29
	LVDs (mm)	5.04 ± 0.74	4.03 -6.48
	LVPWs (mm)	4.51 ± 0.58	3.49 -5.38
	FS (%)	45 ± 6	31 -52
Doppler	MV E Vel (cm/s)	71 ± 9	52 -89
	MV A Vel (cm/s)	57 ± 11	43 -81
	E/A	1.34 ± 0.24	1.01 -1.74
	IVRT (msec)	34 ± 4	26 -39
	AV max (cm/s)	77 ± 10	57 -90
	PV max	87 ± 11	70 -105

High sensitivity cTnT concentrations were measured in seven squirrel monkeys with a mean ± SD of 0.049 ng/m ± 0.043 ng/mL and a range of 0.005 -0.129 ng/L. NT -proBNP concentrations in these seven squirrel monkeys were all below the detection limit of 0.5912 pmol/L. A summary of the characteristics of the included squirrel monkeys and adjacent cTnT values and echocardiographic findings can be found in Table 2.

**Table 2:** Summary of included Squirrel monkeys (species, sex, age, weight) with concurrent cTnT levels and relevant echocardiographic findings (MI: mitral valve insufficiency; AI: aortic valve insufficiency; TI: tricuspid valve insufficiency; PI: pulmonic valve insufficiency)

Subspecies	Sex	Age	Weight (g)	Cardiac Troponin T (ng/L)	Echocardiographic findings
**Sciureus**	**Female**	**18**	**752**	**59.85**	**Trivial PI**
**Sciureus**	**Female**	**6.3**	**756**	**14.31**	**Trivial PI, TI**
**Sciureus**	**Female**	**18.6**	**740**	**76.42**	**Trivial PI, TI**
**Boluviensis peruviensis**	**Female**	**4.2**	**503**	**5.01**	**Trivial PI**
**Boluviensis peruviensis**	**Female**	**21.3**	**534**	**129.3**	**Mild MI, TI**
**Boluviensis peruviensis**	**Female**	**6.2**	**614**	**17.37**	**Trivial PI, TI**
**Sciureus**	**Female**	**18.6**	**681**	**41.9**	**Trivial TI**
**Boluviensis peruviensis**	**Female**	**4.25**	**598**		**Trivial TI**
**Boluviensis peruviensis**	**Female**	**5.2**	**574**		**Trivial AI**
**Boluviensis peruviensis**	**Female**	**2.25**	**467**		**Trivial TI**
**Sciureus**	**Female**	**9.2**	**628**		**Trivial PI, TI**
**Boluviensis peruviensis**	**Male**	**2.25**	**530**		**Trivial PI TI**
**Boliviensis peruviensis**	**Male**	**2.25**	**618**		**None**

**Fig. 1:**
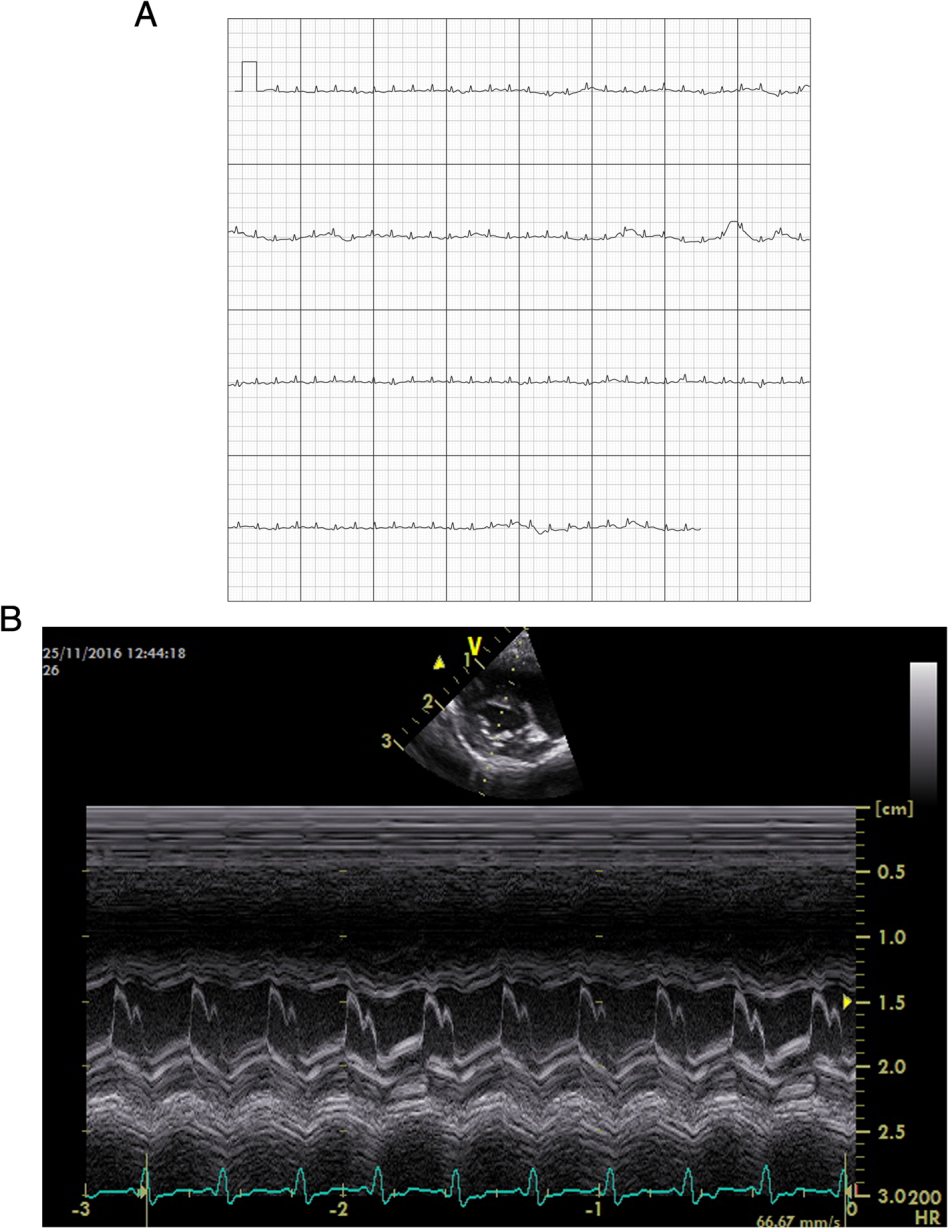
**a** Electrocardiographic examination of a clinically healthy and echocardiographically normal Squirrel monkey recorded by AliveCor Vet TM® (duration: 30s; 50 Hz scale: 25 mm/s, 10 mm/mV). **b** Electrocardiographic examination of a clinically healthy and echocardiographically normal Squirrel monkey recorded by GE Vivid-I portable ultrasound machine (duration: 30s; 50 Hz scale: 25 mm/s, 10 mm/mV). This is the same monkey as Fig. 1a in order to compare the quality of the electrocardiographic recordings of both the AliveCor Vet TM® and GE Vivid-I portable ultrasound machine

Electrocardiographic examination was performed in all but one *Saimiri boliviensis*, due to technical difficulties. The mean heart rate ± SD in these 12 squirrel monkeys (*Saimiri* spp.) during the echocardiographic examination was 172 ± 40 bpm (121 -236 bpm). No cardiac arrhythmias were detected (Fig. 1).

## Discussion

This study shows that qualitative transthoracic echocardiographic images can be obtained in squirrel monkeys using imaging planes and a scanning protocol similar to canine and feline echocardiography. Furthermore, normal echocardiographic values were generated in clinically healthy squirrel monkeys and to the authors’ knowledge, this study is the first to report 25 echocardiographic measurements in this species, adding 18 additional measurements compared to previous research [12, 26]. Additionally, serum concentrations of cTnT in 7 squirrel monkeys are reported and NT -proBNP results were below detection limit in this healthy cohort.

Despite the fact that cardiomyopathy is among the most common causes of death in aging squirrel monkeys (*Saimiri* spp.), there is little data available regarding echocardiographic evaluation of the heart in this particular species [12, 26]. Huss et al. [26] evaluated both electrocardiographic and echocardiographic values in 63 clinically healthy squirrel monkeys. In the study of Huss et al. [26], the animals were not sedated for the actual echocardiographic examination, however they were sedated with ketamine 1 week after echocardiographic assessment to perform the electrocardiographic examination. The latter study included 2D and M -mode echocardiography which was performed in non -sedated monkeys, which were handheld in left lateral recumbence. Echocardiography was performed with the transducer in left parasternal short -axis view to determine the left LVIDs, LVIDd and EF. How the latter value was calculated was however not described in that study. In the study of Brady et al. [12], the echocardiographic examination was performed with animals in a dorsal recumbency, anesthetized with a combination of ketamine and xylazine, measuring LVIDs, LVIDd, LVSAXs, LVSAXd, LADd and Ao. The results of Brady et al. [12] and Huss et al. [26] are similar to the measurements obtained in this study, although the animals in our study group were anaesthetized with a different protocol. Multiple studies have described an influence of different anaesthetic protocols on echocardiography in several species, including a significant decrease of LVIDd, IVSs and FS in dogs anaesthetized with isoflurane [41–46]. In this case, it seems that the effect of short term isoflurane inhalation anesthaesia seems to be minimal.

**Fig. 2:**
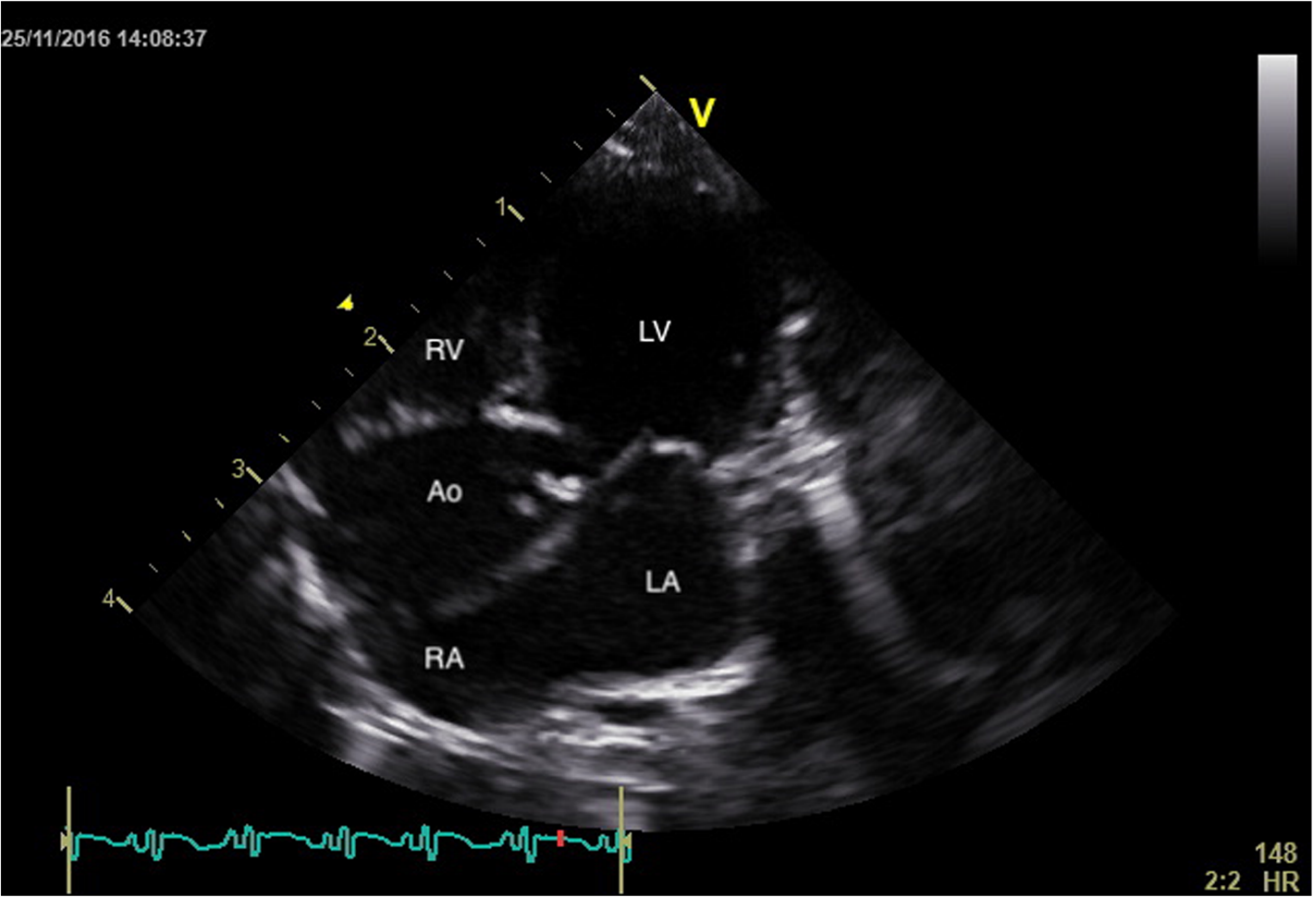
Two-dimensional echocardiographic apical five chamber view of a clinically normal Squirrel monkey with severe aortic dilation (LA: left atrium; RA: right atrium; RV: right ventricle; Ao: aorta)

**Fig. 3:**
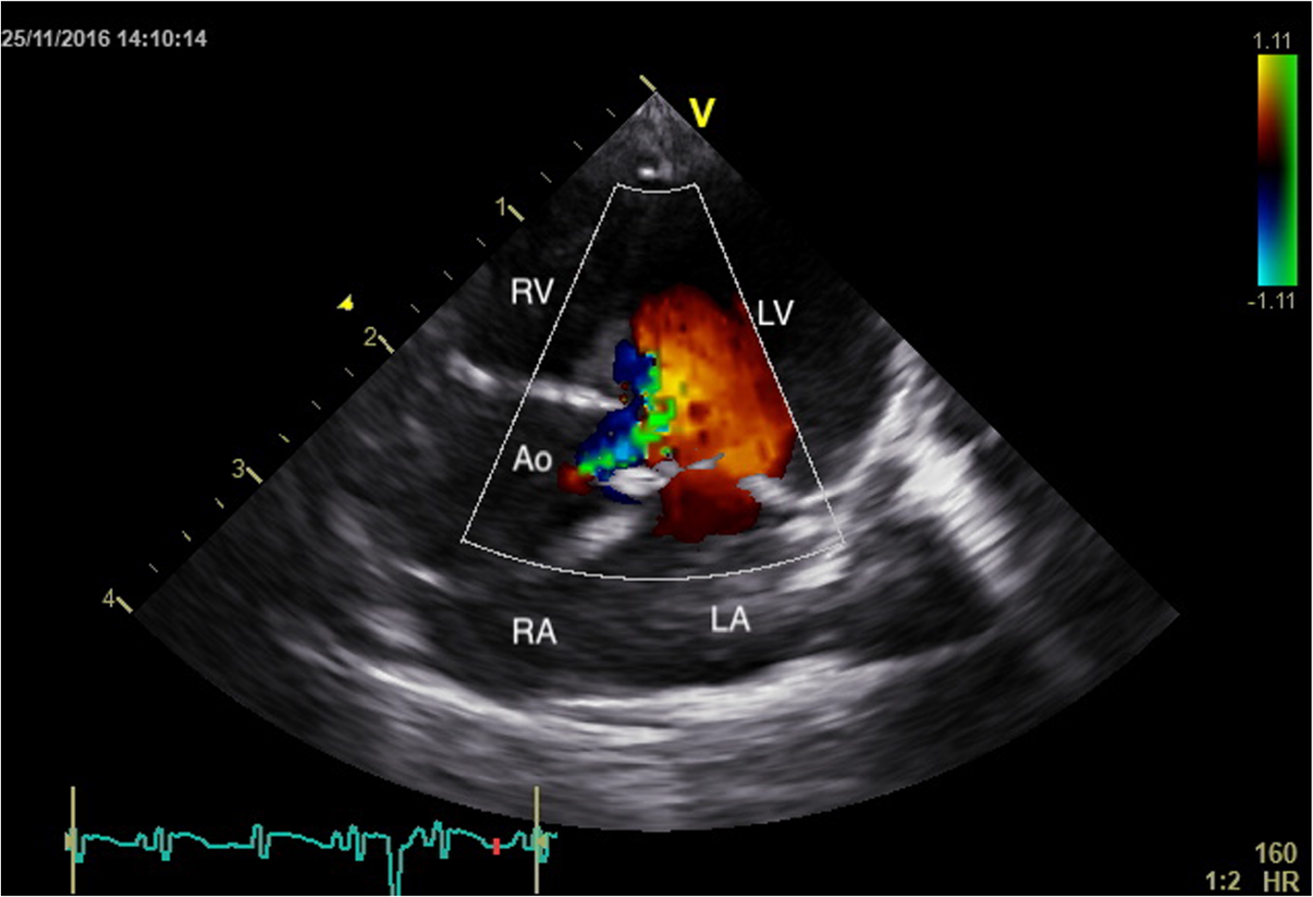
Colour doppler mode of the aortic valve flow in the echocardiographic apical five chamber view of a clinically normal Squirrel monkey with severe aortic dilation. Moderate aortic regurgitation is visible (LA: left atrium; RA: right atrium; RV: right ventricle; Ao: aorta)

This study shows that TTE represents a useful, non -invasive imaging technique for antemortem evaluation of cardiac structure and function. Furthermore, TTE could also be a valuable tool for performing cardiovascular physiological studies and/or non -invasive routine cardiac screening in squirrel monkeys (*Saimiri* spp.). The relevance of this statement was proven by the fact that one apparently healthy squirrel monkey was diagnosed with significant aortic dilation and insufficiency and frequent supraventricular premature beats (Figs. 2 and 3). Cardiac biomarkers were unfortunately not determined in this squirrel monkey. Conclusively, the echocardiographic normal values obtained in this study could function as the stepping stone for future studies in the squirrel monkey (*Saimiri* spp.).

Regarding the ECG data, the mean heart rate in our study was significantly lower compared to the study of Huss et al. [26], namely 172 bpm compared to 280 bpm, though similar to the results of Brady et al. [12], namely 191 bpm. The difference with the study of Huss et al. [26] might be attributed to the level of consciousness secondary to the light sedation which was used during electrocardiographic examination compared to the general anaesthesia which was applied in our study and the study of Brady et al. [12]. In addition, previous studies have shown that ketamine, which was applied in the study of Huss et al. [26], might increase the heart rate, although a study by Gonder et al. [47] has shown that the latter does not significantly influence the ECG findings in primates. Ketamine was used in the study of Brady et al. [12], with similar electrocardiographic results, supporting the results of Gonder et al. [47].

Additionally, cardiac biomarkers cTnT and NT -proBNP were determined. To the best of the authors’ knowledge, this is the first study evaluating the cTnT or NT -proBNP values in squirrel monkeys (*Saimiri* spp.). In healthy humans and animals, both circulating cTnT and NT -proBNP values are low. As myocardial cells are damaged secondary to inflammation or infarction, cTnT, which is tightly bound to sarcomeric actin filaments under physiological circumstances, leaks into the cytosol and interstitial space. Both in human and veterinary medicine, cTnT has proven its diagnostic value in evaluating the presence and severity of myocardial damage. Given its high conservation across species, we expected it will be of value in squirrel monkeys as well [31, 32, 48–54]. Previous studies have shown that, apart from cardiomyopathies, cTnT can be elevated secondary to non -cardiac causes, such as renal failure [50]. Theoretically, the presence of non -cardiogenic, subclinical pathologies which might influence the levels of cTnT cannot be excluded based on the performed tests, although this is unlikely given the clinical healthy condition of all included squirrel monkeys.

NT -proBNP is the prohormone of B -type natriuretic peptide (brain natriuretic peptide, BNP). It will become elevated secondary due to myocardial stress, which can be caused by multiple cardiomyopathies. Given its longer circulating half -life, higher concentration and more stable nature in stored (frozen) plasma or serum compared to BNP, NT -proBNP is preferred as a cardiac biomarker over the latter [55–57]. Both in human and veterinary medicine, NT -proBNP has proven its diagnostic value in order to distinguish cardiogenic from respiratory causes of dyspnea, evaluate and monitor the presence, severity and progression of cardiac disease [58–60]. Human and veterinary studies have shown that, apart from cardiomyopathies, NT -proBNP can be elevated secondary to renal failure and inflammation [61–63]. As there is a significant interspecies, and in veterinary medicine, even inter -breed variation of NT -proBNP reference values cannot be extrapolated between species. Therefore it should be attempted to establish normal values for each individual species, including the squirrel monkey [64, 65]. NT -proBNP in our study was below the detection limit of 0.5912 pmol/L. The possible interpretation of these results is twofold. Either clinically healthy squirrel monkeys (*Saimiri* spp.) have low NT -proBNP values or the human NT -proBNP test used in this study is not applicable in this species. Recent research in Cynomologous monkeys has shown that caution should be used with the interpretation of NT -proBNP when applying human assay kits as good cross reactivity was not observed despite 90% sequence homology of NT -PROBNP between humans and monkeys [66].

## Limitations

As is often the case in zoological studies and because this study was limited to the zoo colony of Pairi Daiza, the low number of squirrel monkeys included is a limitation of the study. The sample size does not allow an assessment of statistical differences in echocardiographic variables between male and female monkeys or young and older animals. Furthermore, given the low number of squirrel monkeys and the fact that all NT -proBNP concentrations were below detection limit, this study was not able to establish reference values for cTnT nor NT -proBNP. Despite these limitations, we were able to demonstrate that a standard veterinary echocardiographic scanning protocol can be used to generate an extensive set of echocardiographic normal values and described it in detail. Nonetheless, additional studies with a larger number of animals allowing differentiation between male versus female monkeys and also young versus older animals are necessary in the future.

## Conclusion

This study showed that qualitative echocardiographic images can be obtained in squirrel monkeys, following an echocardiographic scanning protocol similar as has been described in canine and feline cardiology. Secondly, normal values for 2D, M -Mode and spectral Doppler measurements were established. Lastly, to the best of our knowledge, this is the first study to report normal values of cTnT in squirrel monkeys (*Saimiri* spp.), whereas concentrations of NT -proBNP were below detection limit. Results of this study are valuable in zoological medicine as well as in future research involving non -human primate models of cardiovascular disorders and morphologic studies on squirrel monkeys (*Saimiri* spp.).

## Methods

The annual health examination of the squirrel monkey colony consisted of a physical examination, blood sampling, thoracic radiography and TTE. These were performed under general anesthesia on site in the veterinary hospital of Pairi Daiza zoo, Brugelette, Belgium. All examinations were performed in adherence to the guidelines of the Weatherall report on the use of non -human primates and according to Directive 210/63/EU [25].

### Animals

Fourteen captive squirrel monkeys (Saimiri spp.), 9 *Saimiri boliviensis peruviensis* and 5 *Saimiri scireus*, from and owned by Pairi Daiza, Belgium were included in the study and underwent physical examination, blood sampling, thorax radiography and TTE performed by a veterinary cardiologist (PS). A description of the thoracic radiographic findings of this cohort of monkeys has been previously published [25]. Monkeys were housed and fed as described earlier in the study of Houdellier et al. [25].

### Anaesthesia

The general health examination was the sole reason of the general anesthesia in all included animals. General anesthesia, including both induction and maintenance, was by mask. To minimize resistance and dead space, an Ayre’s T -piece was used. The volume of oxygen was fixed at 2 L/min at all times and the percentage of isoflurane (Isoflo, Ecuphar) adjusted to the anaesthetic monitoring of the patient by checking eyelid (medial palpebral reflex) and cornea reflexes. The percentage was on the maximum value (5%) for induction and was steadily reduced for maintenance until 1,5 ± 2%. Twenty milliliter of saline solution (Ringer Lactate1 solution) was administered subcutaneously to all animals at the beginning of the procedure. Throughout the course of the complete anaesthesia, respiratory rate was counted visually, heart rate and oxygen saturation were monitored using pulse -oxymetry placed on the hand and rectal temperature was continuously monitored with a digital probe. In order to maintain the latter, gloves were filled with hot water (37 °C), a heating carpet (38 °C) and a pre -heated sonographic gel were used. After completion of the general health examination, animals were recovered in a small dark transport cage in a heated room and transported back to their enclosure thereafter. Animals were monitored after anaesthesia for 3 consecutive days. No adverse effects were recorded [25].

A complete echocardiographic examination was performed by a board -certified cardiologist and an electrocardiogram (ECG) with the Alivecor system (AliveCor Vet TM®) after the radiographic thoracic examination. The mean time of anesthesia was 31 min, ranging from 22 min to 35 min [25].

### NT -proBNP and cardiac troponin T

Blood samples were taken in seven squirrel monkeys by femoral venipuncture and collected in 4 -mL blood tubes, EDTA (ethylenediaminetetraacetic acid) and serum separator tubes. The remaining 6 squirrels monkeys could either not be sampled or blood sample volume was insufficient. Samples were centrifuged within 30 min, plasma and serum samples were stored at 4 °C until testing. cTnT serum and NT -proBNP concentrations were determined using the chemiluminescent sandwich ELISA human high sensitive (hs) Troponin T (Cobas®) and human proBNP II (Cobas®) tests, respectively.

### Echocardiography

**Fig. 4:**
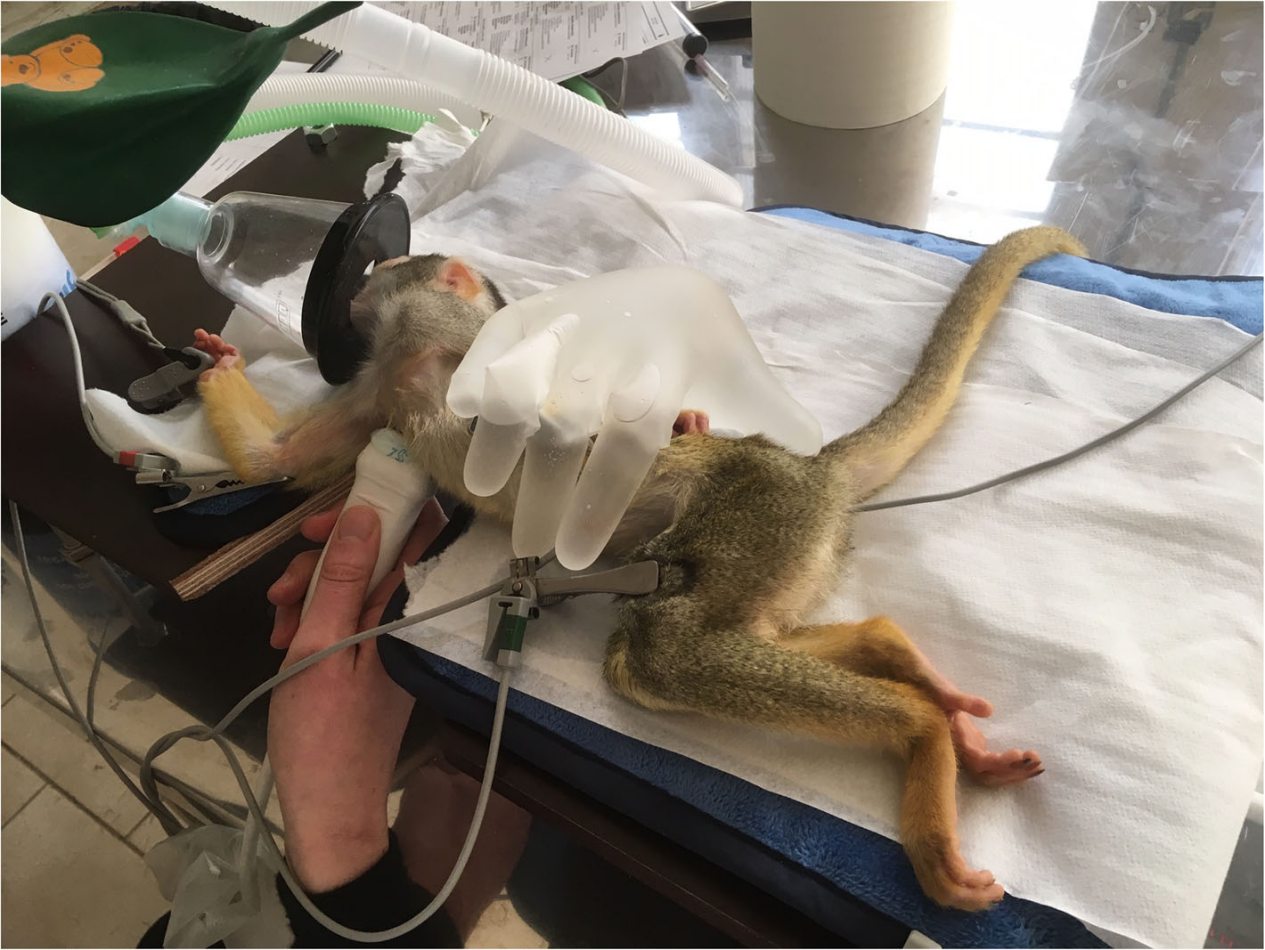
Squirrel monkey positioned in right lateral recumbency during echocardiographic and electrocardiographic examination. A right parasternal long-axis four-chamber image was obtained by placing the transducer within the 3rd to 6th intercostal space, close to the sternum, with the reference mark toward the neck and a 45° angle between the transducer and the squirrel monkey

Echocardiographic examinations were performed by a single operator (PS) using a GE Vivid -I portable ultrasound machine and a 3.5 -7.5 MHz phased array transducer (settings: frame rate: 77 frames per second; power: -2 dB; depth: 3.0 cm (Colour Doppler: frame rate: 106 frames per second; gain: -10 dB; scale: 8.00 kHz; frequency: 3.6 MHz; sample volume: 0.65 mm)) and analysed offline with GE EchoPAC software®. A detailed step -by -step operation procedure of the echocardiographic examination has been added as ‘Supplement I’ to this manuscript and is based on earlier described canine and feline echocardiographic protocols [39, 67]. All squirrel monkeys were examined in right and left lateral recumbency in a quiet, darkened room under general anaesthesia. Animals were positioned on a small custom -made echocardiography table with a cut -out for positioning of the ultrasound probe, on an electrical heating pad with additional warm gloves put against their bodies. Preheated ultrasound gel was used and a rectal temperature probe was inserted to monitor core body temperature (Fig. 4).

**Fig. 5:**
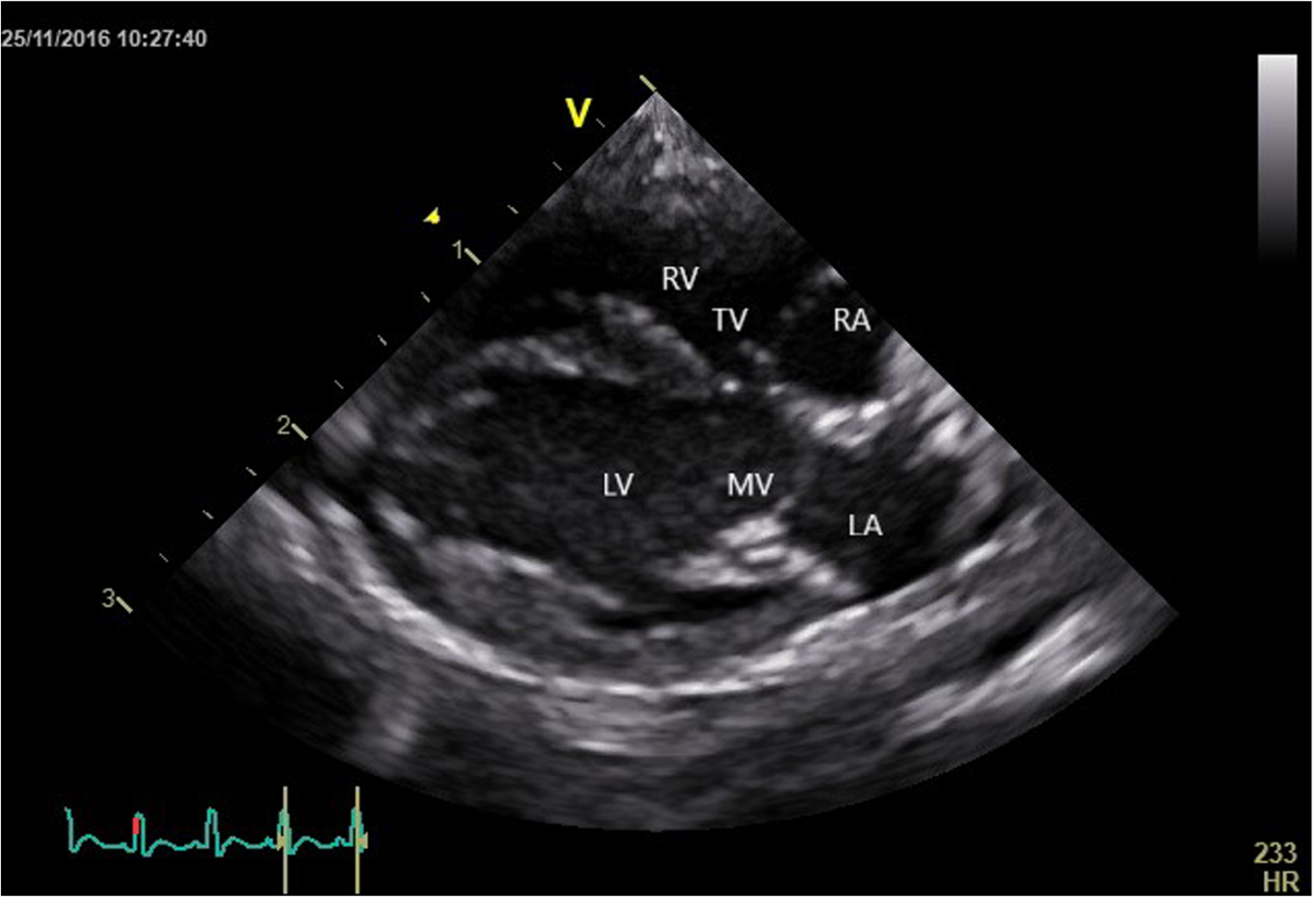
Two-dimensional echocardiographic right parasternal four chamber view of a normal Squirrel monkey (LA: left atrium; LV: left ventricle; MV: mitral valve; RA: right atrium; RV: right ventricle; TV: tricuspid valve)

**Fig. 6:**
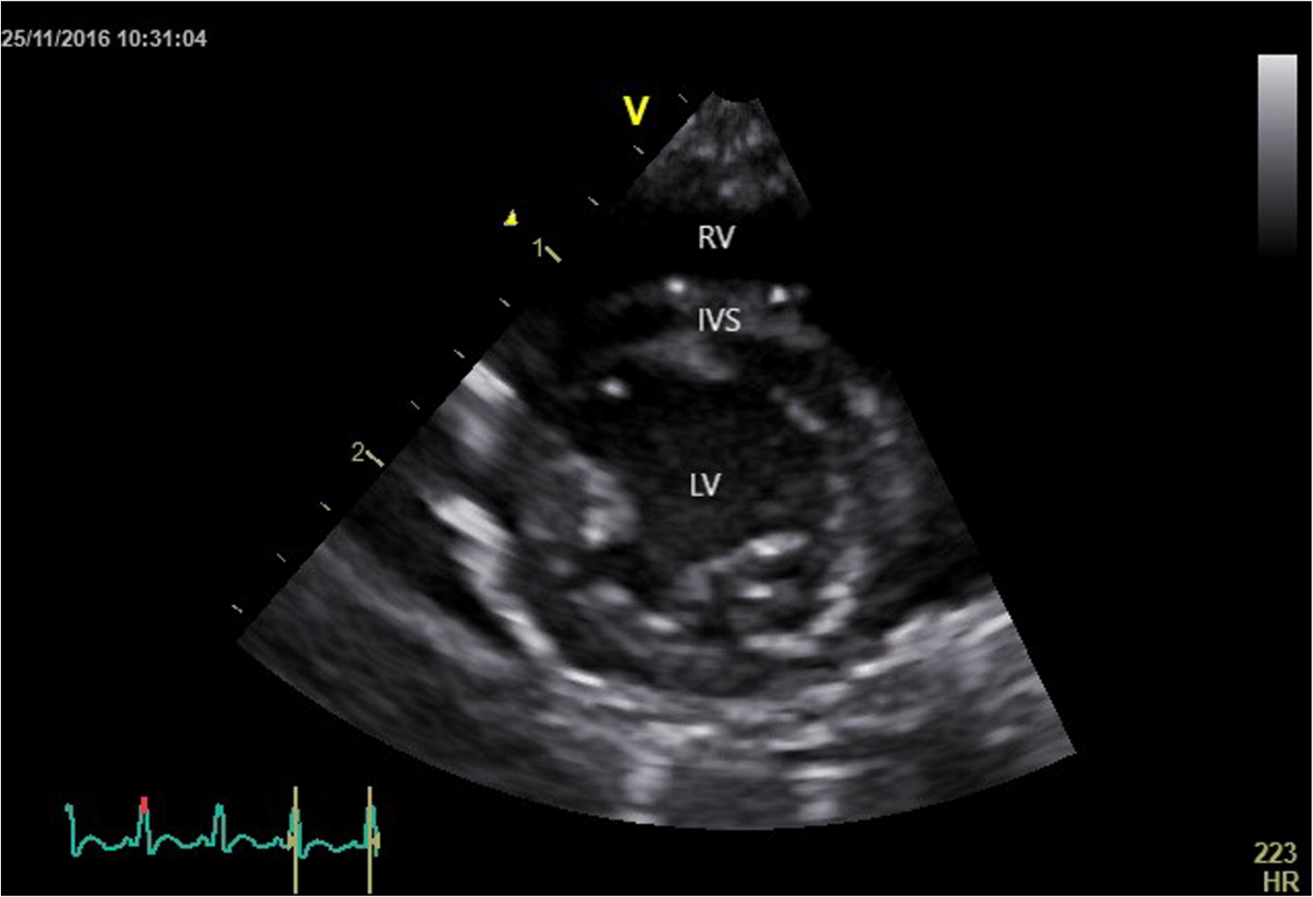
Two-dimensional echocardiographic right parasternal short-axis view at the level of the papillary muscles of a normal Squirrel monkey (LV: left ventricle; RV: right ventricle; IVS: interventricular septum)

**Fig. 7:**
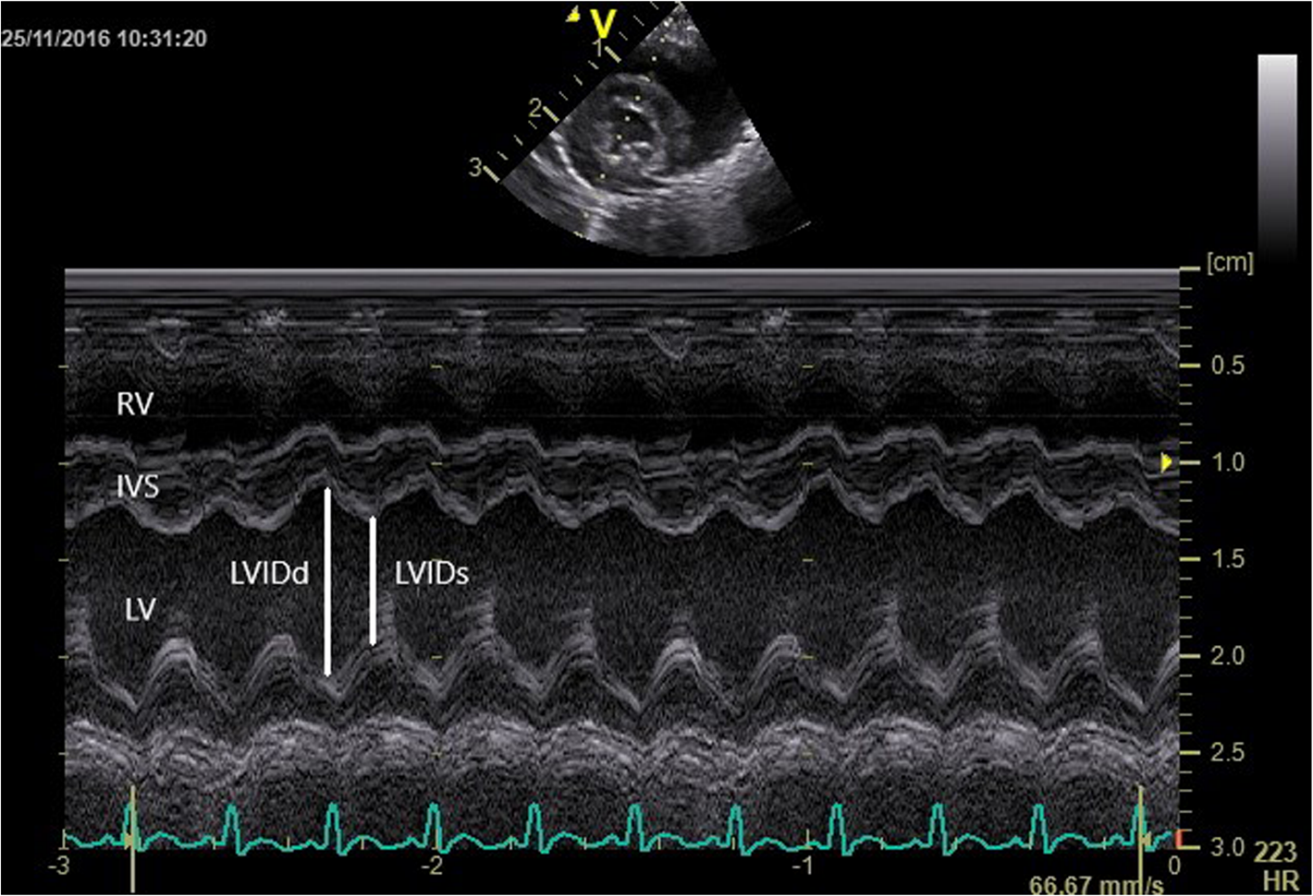
Time-motion echocardiographic right parasternal short-axis view at the level of the papillary muscles of a normal Squirrel monkey (LV: left ventricle; RV: right ventricle; IVS: interventricular septum; LVIDd: internal diameter of the left ventricle during diastole, LVIDs: internal diameter of the left ventricle during systole)

**Fig. 8:**
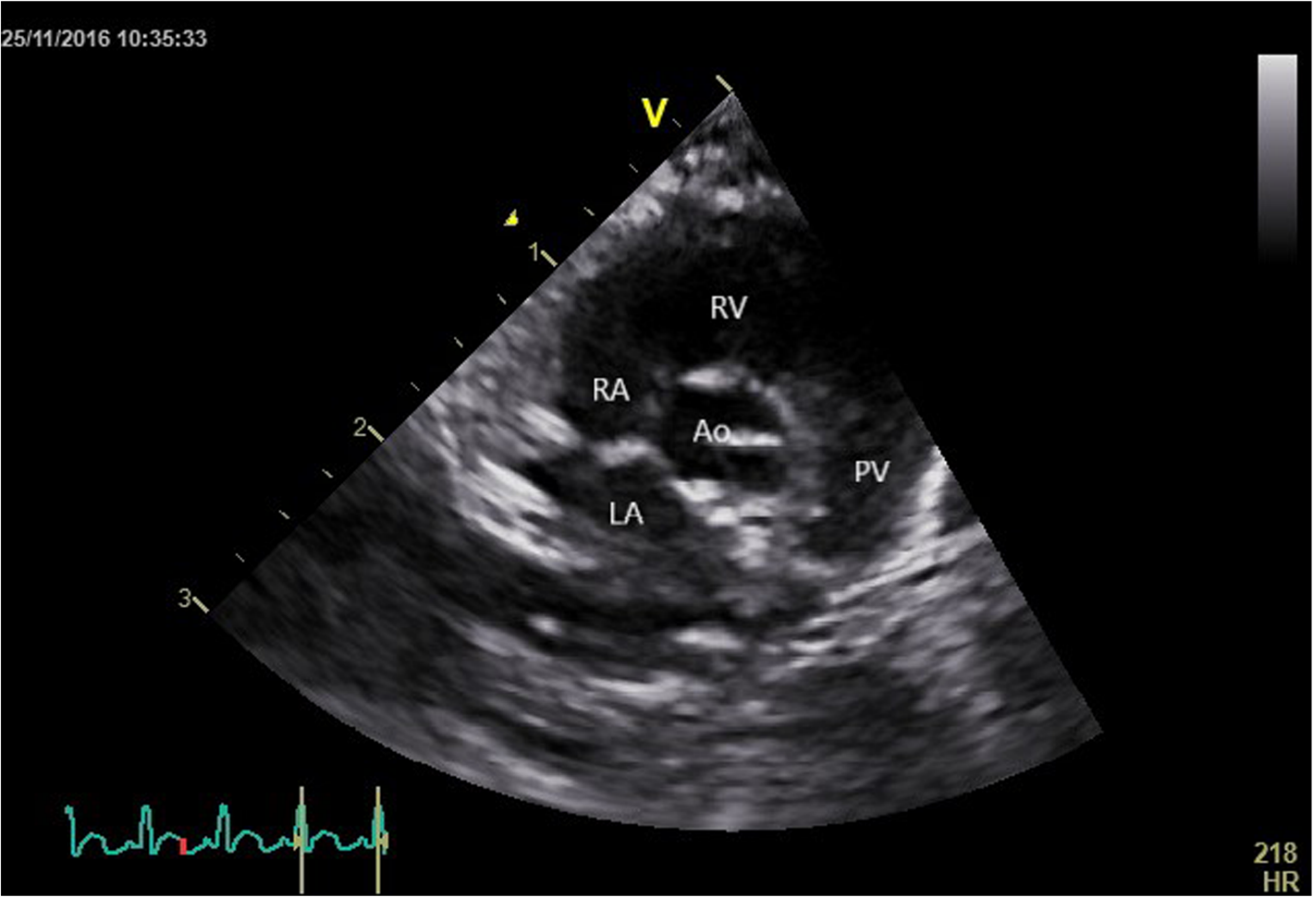
Two-dimensional echocardiographic right parasternal four chamber view at the level of the heart base of a normal Squirrel monkey (LA: left atrium; RA: right atrium; RV: right ventricle; PV: pulmonic valve; Ao: aorta)

The right parasternal long -axis four -chamber image was obtained by placing the transducer within the 3rd to 6th intercostal space, close to the sternum, with the reference mark toward the neck and a 45° angle between the transducer and the squirrel monkey (Figs. 4 and 5). Optimal visualization of the atria was obtained by sliding the transducer within the same intercostal space. The left ventricular outflow tract view could be visualised by rotating the transducer counter clockwise. By continuing this movement until the reference mark was turned approximately 90° from its location from the long -axis four -chamber image and dropping the transducer slightly, the right parasternal short -axis could be obtained (Figs. 6 and 7). Finally, the heart base could be visualised by starting from the latter position, tilting the transducer such that the transducer crystals point even more toward the neck (Fig. 8). Measurements were obtained by applying 2D, M -Mode and spectral Doppler mode.

**Fig. 9:**
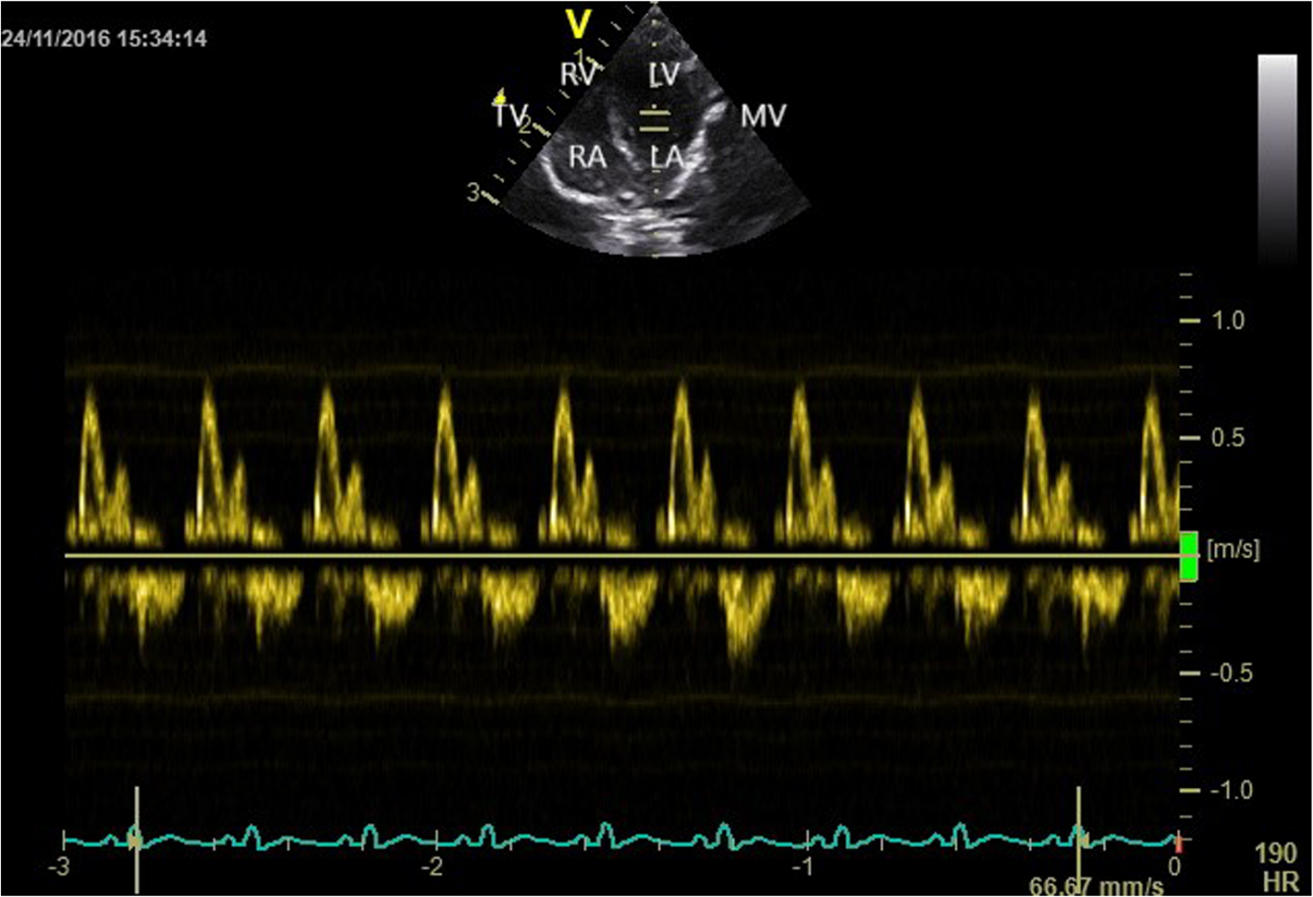
Spectral doppler mode of the transmitral valve flow in the left apical four chamber view of a normal Squirrel monkey (LA: left atrium; LV: left ventricle; MV: mitral valve; RA: right atrium; RV: right ventricle; TV: tricuspid valve)

**Fig. 10:**
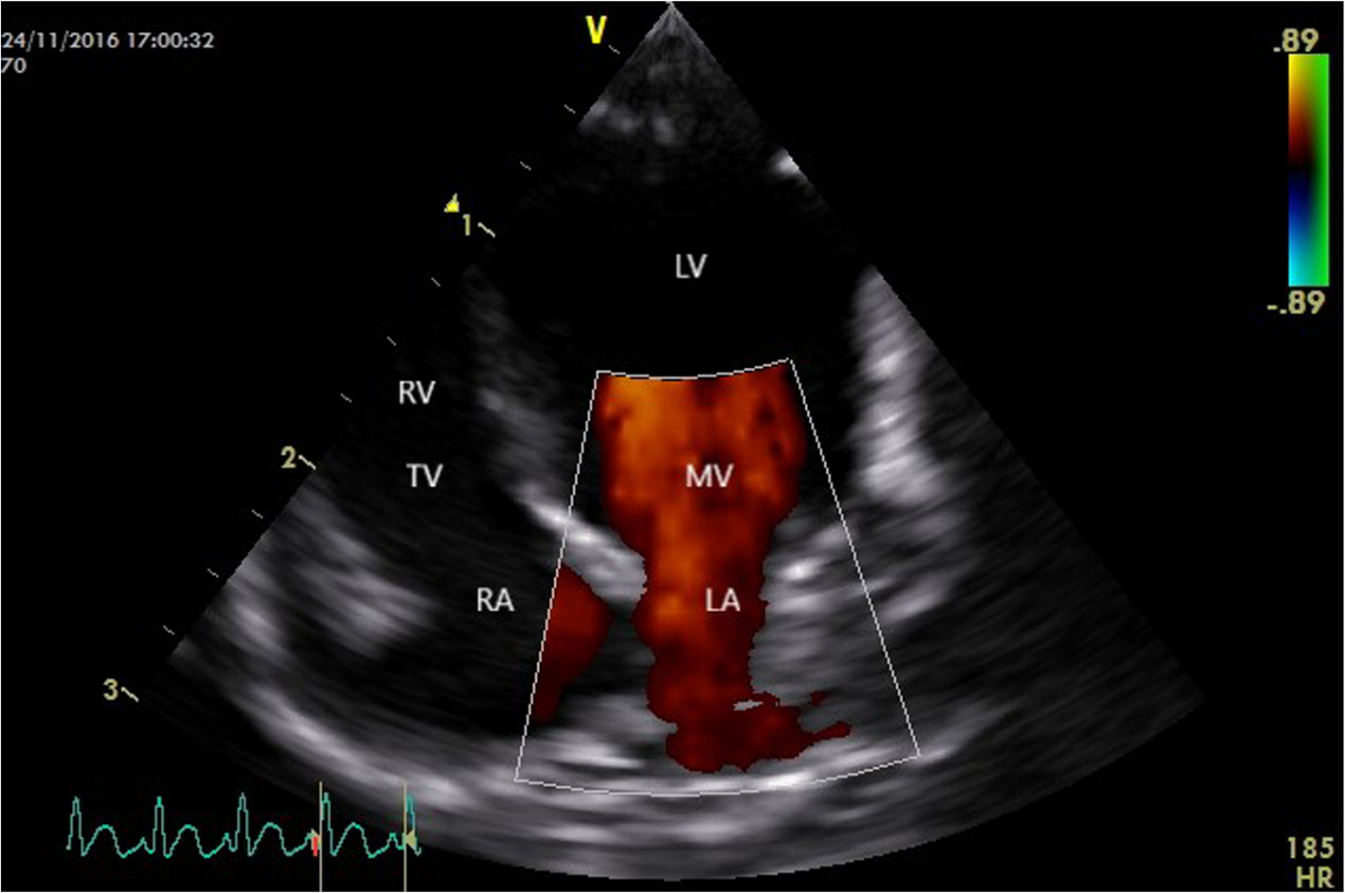
Colour doppler mode of the transmitral valve flow in the left apical four chamber view of a normal Squirrel monkey (LA: left atrium; LV: left ventricle; MV: mitral valve; RA: right atrium; RV: right ventricle; TV: tricuspid valve)

The right apical four -chamber long -axis image was generated with the sound plane cranially along the heart, the transducer placed near the apex of the heart, starting close to the xyphoid, moving cranially along the sternum and the crystals directed toward the shoulder and base of the heart. Whilst remaining in the same intercostal space, the left ventricular outflow tract could be visualised by slightly rotating the transducer. The transvalvular flow of the atrioventricular valves and aortic velocity were evaluated by applying spectral Doppler mode (Fig. 9). Mitral -, tricuspid -, pulmonic and aortic valves were evaluated for the presence of valvular insufficiencies using color flow Doppler in multiples imaging planes (Fig. 10).

### Electrocardiography

Immediately before echocardiography an electrocardiogram (ECG) was recorded via a commercial, non -invasive, smartphone -based monitoring device (AliveCor Vet TM®). Similar to the use of the AliveCor Vet TM® in cats, the hands of the monkeys were placed at the contact pads. Electrocardiograms were recorded in right lateral recumbency for at least 30 s by using a chart speed of 25 mm/s whilst the gain was adjusted for 10 mm of deflection per millivolt. Electrical contact was enhanced by the application of alcohol. During the echocardiographic examination, ECG was also performed, using live monitoring on the GE Vivid -I portable ultrasound machine. Figure 1a and b show the electrocardiographic recordings of the same monkey obtained by both the AliveCor Vet TM® and GE Vivid -I portable ultrasound machine.

### Statistics

All variables were evaluated visually for normality using Q -Q plots. As all variables were normally distributed, the mean and standard deviation (SD) are given, together with the range. Only animals considered healthy were included when the species -specific normal values are reported. The statistical analysis was conducted in R version 3.4.4.

## Supplementary information

**Additional file 1.** Supplement I: Operating procedure of the echocardiographic examination in healthy captive male and female squirrel monkeys.

## Data Availability

The datasets during and/or analysed during the current study are available from the corresponding author on reasonable request.

## Abbreviations

2D: Two-dimensional
BPM: Beats per minute
LV: Left ventricle/left ventricular
M-mode: Time -motion mode
TTE: Transthoracic echocardiography
SD: Standard deviation
IVSd: Interventricular septal thickness at end -diastole
IVSs: Interventricular septal thickness at end -systole
LVIDd: Left ventricular internal dimension at end -diastole
LVIDs: Left ventricular internal dimension at end -systole
LVPWd: Left ventricular posterior wall thickness at end -diastole
LVPWs: Left ventricular posterior wall thickness at end -systole
FS: Fractional shortening
Ao: Aortic root diameter
LAD: Left atrial diameter
LA:Ao: Ratio of the left atrial dimension to the aortic annulus dimension
LAloAx: Left atrium long axis
RAloAx: Right atrium long axis
MV E Vel: Transmitral valve flow Ewave velocity
MV A Vel: Transmitral valve flow A -wave velocity
E/A ratio: Transmitral valve flow E -wave to mitral valve flow A -wave ratio
IVRT: Isovolumetric relaxation time
AV max: Aortic valve maximal flow velocity
PV max: Pulmonic valve maximal flow velocity
cTnT: Cardiac troponin T
NT -proBNP: N -terminal pro -brain natriuretic peptide
