# Supplementary material for: Transthoracic echocardiography and cardiac biomarkers in healthy captive male and female squirrel monkeys (Saimiri spp.)
Source: BMC Vet Res. 2020 Jun 29;16:217. doi: 10.1186/s12917-020-02406-3 (PMC7322930; doi:10.1186/s12917-020-02406-3)
Supplement: Supplementary file 1 — Additional file 1. Supplement I: Operating procedure of the echocardiographic examination in healthy captive male and female squirrel monkeys. [file 12917_2020_2406_MOESM1_ESM.docx]

**Supplement I: Operating procedure of the echocardiographic examination in healthy captive male and female squirrel monkeys.**

| **View** | **Probe Position** | **Image** | **Uses** |
| --- | --- | --- | --- |
| **Right parasternal long-axis four chamber** | The right parasternal long-axis four-chamber image can be obtained by placing the transducer within the 3rd to 6th intercostal space, close to the sternum, with the reference mark toward the neck and a 45° angle between the transducer and the squirrel monkey. Optimal visualization of the atria is obtained by sliding the transducer within the same intercostal space. Measurements can be obtained by applying 2D, M-Mode. | 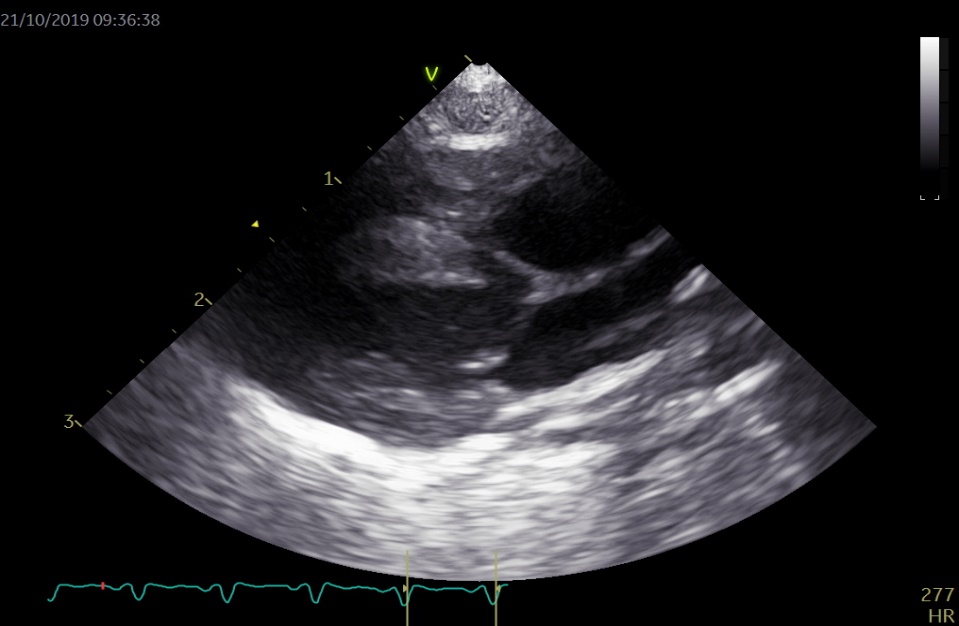    RV  RV  RA  jfld  LA  LVV | ***2D:**  Overview of all four chambers  LA and RA dimensions  LV dimensions  Mitral valve morphology and motion  ***Colour Doppler:**  Mitral valve regurgitation  Tricuspid valve regurgitation |
| **Right parasternal long-axis five chamber** | The left ventricular outflow tract view can be visualised by rotating the transducer counter clockwise. Measurements can be obtained by applying 2D and M-Mode. | 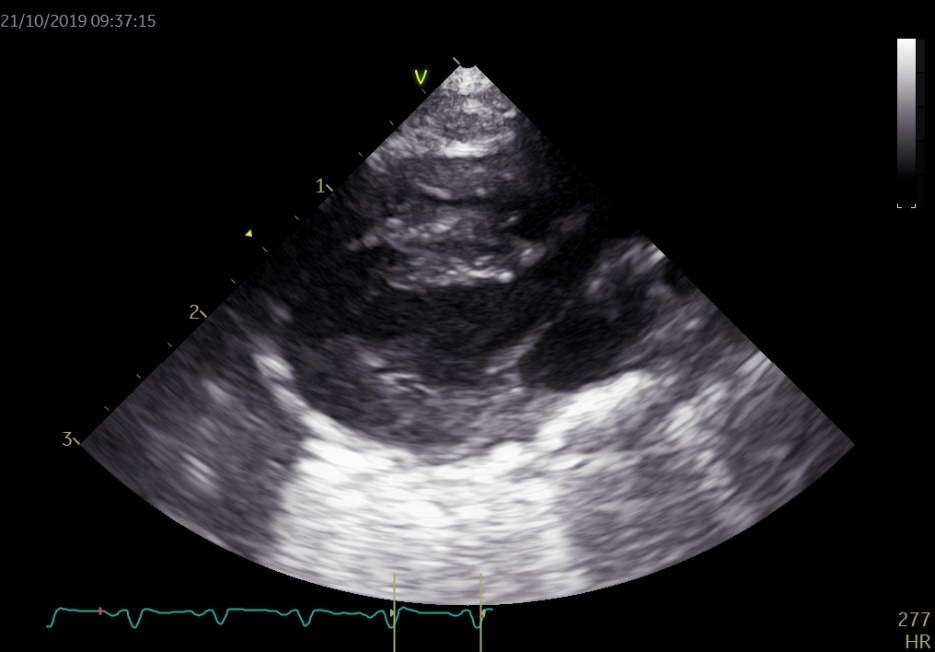  Ao  LA  LV | ***2D:**  LVOT abnormalities  LV wall thickness  ***Colour Doppler:**  LVOT obstruction  Aortic regurgitation  Mitral regurgitation |
| **Right parasternal short-axis (papillary muscle level)** | By continuing this movement until the reference mark was turned approximately 90° from its location from the long-axis four-chamber image and dropping the transducer slightly, the right parasternal short-axis can be obtained. Measurements can be obtained by applying 2D and M-Mode. | 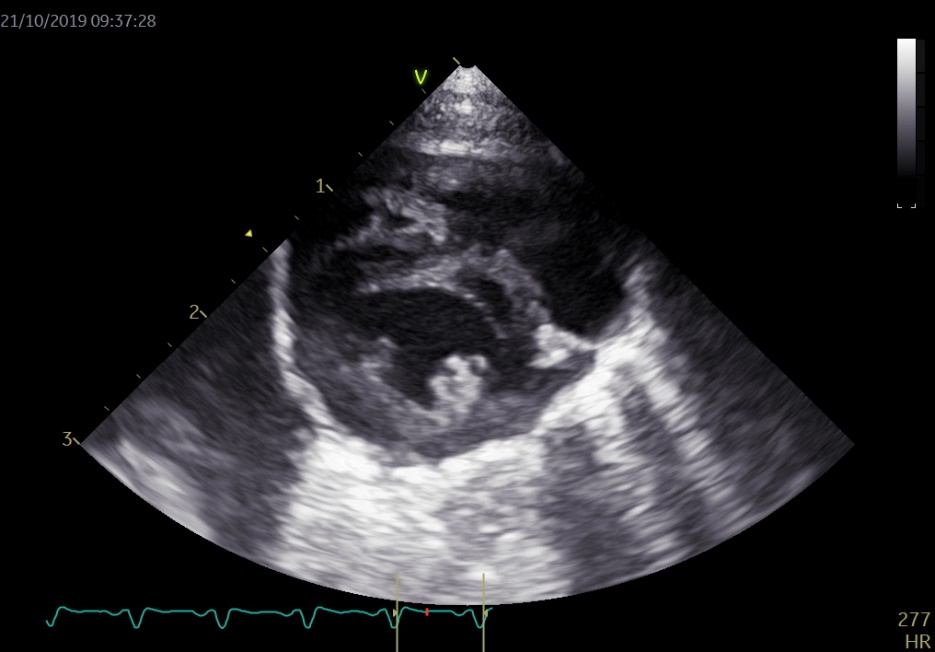  RV  LV | ***2D:**  LV dimensions  ***M-mode:**  LV dimensions |
| **Right parasternal short-axis (aortic valve level)** | The heart base can be visualised by starting from the latter position, tilting the transducer such that the transducer crystals point even more toward the neck. Measurements can be obtained by applying 2D and spectral Doppler mode | 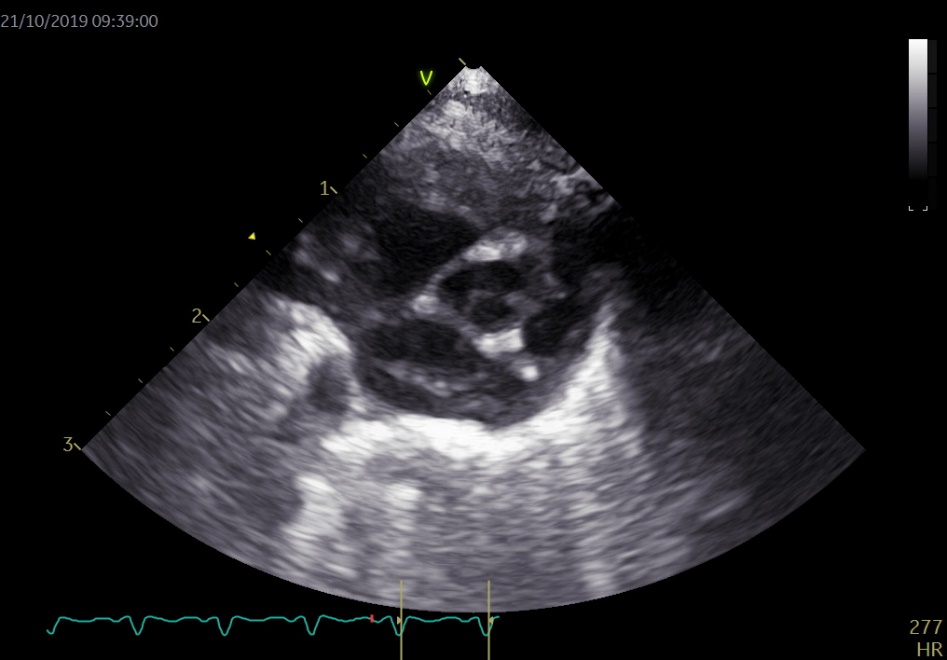  PA  Ao  RA  LA | ***2D:**  LA and aortic dimensions  Aortic valve morphology  Tricuspid valve morphology  ***Colour Doppler:**  Valvular insufficiencies |
| **Right parasternal short-axis (pulmonary artery level)** | Once a good image of the heart base with aorta and left atrium, the probe is tilted toward the heart base in order to bring the main pulmonary artery into view. | 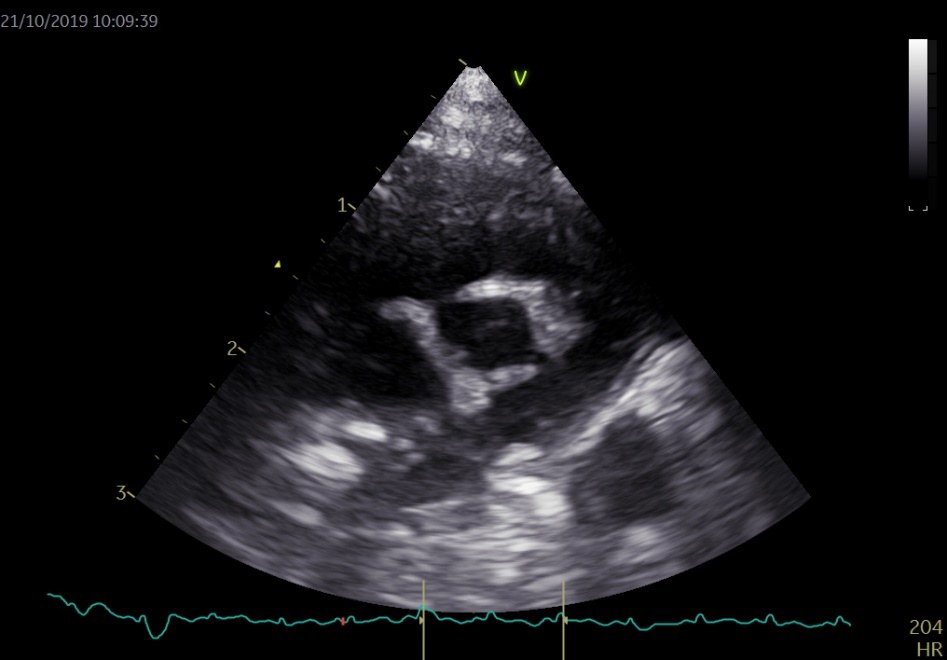  RV  RA  Ao  PA  LA | ***2D:**  RVOT/pulmonary valve morphology  ***Colour Doppler:**  Pulmonary regurgitation  Pulmonary artery blood flow  ***Spectral Doppler:**  Pulmonary artery blood flow velocity |
| **Left apical four chamber** | The left apical four-chamber long-axis image can be generated with the sound plane cranially along the heart, the transducer placed near the apex of the heart, starting close to the xyphoid, moving cranially along the sternum and the probe directed toward the shoulder and base of the heart. | 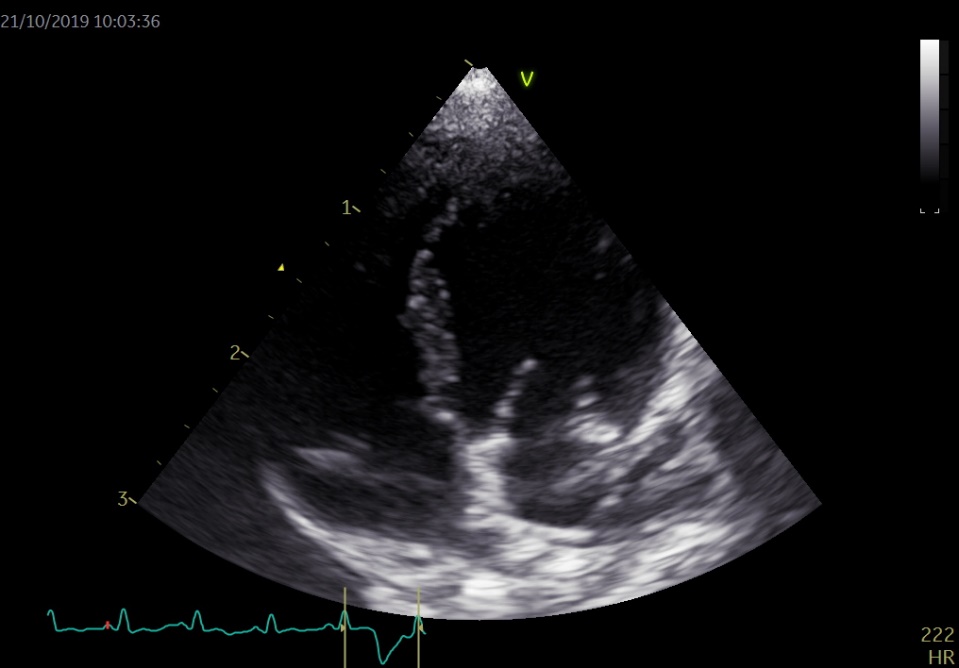  RA  RV  LA  LV | ***2D:**  Overview of all four chambers  Mitral and tricuspid valve morphology and function  ***Colour Doppler:**  Mitral regurgitation  Tricuspid regurgitation  ***Spectral Doppler:**  Mitral valve inflow |
| **Left apical five chamber** | Whilst remaining in the same intercostal space, the left ventricular outflow tract can be visualised by slightly rotating the transducer clock-wise. Measurements can be obtained by applying 2D, M-Mode and spectral Doppler mode. | 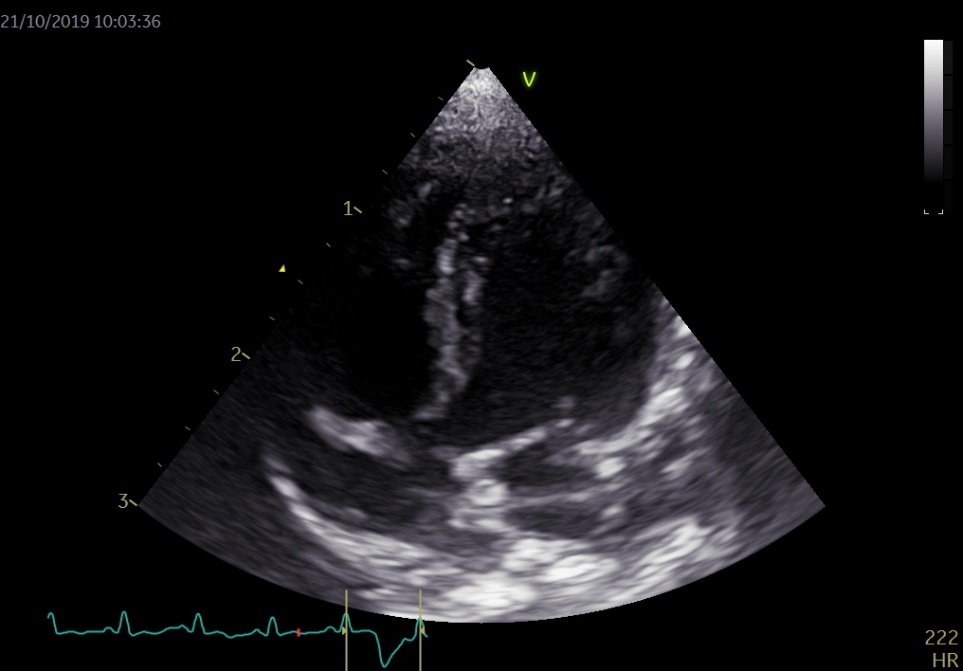  RV  RA  Ao  LA  LV | ***2D:**  LVOT  ***Colour Doppler:**  LVOT obstruction  Aortic regurgitation  ***Spectral Doppler:**  Aortic flow veloticity  Isovolumetric relaxation time |

**Ao: Aorta; AoV: aortic valve; LA: left atrium; LV: left ventricle; LVOT: left ventricular outflow tract; MV: mitral valve; PA: pulmonary artery; RA: right atrium; RV: right ventricle; RVOT: right ventricular outflow tract.**
